# Early diagnosis of tuberculous meningitis by an indirect ELISA protocol based on the detection of the antigen ESAT-6 in cerebrospinal fluid

**DOI:** 10.1007/s11845-013-0980-4

**Published:** 2013-07-18

**Authors:** F. Song, X. Sun, X. Wang, Y. Nai, Z. Liu

**Affiliations:** Department of Neurology, Yantai Yuhuangding Hospital, Yantai, 264000 Shandong Province China

**Keywords:** Tuberculous meningitis, Tuberculosis, Cerebrospinal fluid, ESAT-6, ELISA, Diagnosis

## Abstract

**Background:**

Early diagnosis of tuberculous meningitis (TBM) is still a challenge; the present study aimed to establish a method of detecting the antigen *early secreted antigenic target 6* (ESAT-6) in cerebrospinal fluid (CSF) by an indirect enzyme-linked immunosorbance assay (ELISA) protocol and to study the value of detecting ESAT-6 in CSF in the early diagnosis of TBM.

**Methods:**

An indirect ELISA protocol was used, employing a monoclonal antibody (mAb) against ESAT-6, which was used to demonstrate ESAT-6 in the CSF from TBM patients and non-TBM controls. CSF was obtained from 100 patients: confirmed TBM, clinically diagnosed TBM, disease controls, and healthy controls (*n* = 10). Pure recombinant ESAT-6 (standard product) was used in serial dilutions to detect the absorbance and to establish a standard curve from the data; the concentration was on the *X* axis vs. absorbance on the *Y* axis, and the standard curve was used to interpolate the concentration of ESAT-6 in samples.

**Results:**

The indirect ELISA method provided 88 % sensitivity and 92 % specificity for the diagnosis of TBM using a mAb to ESAT-6. The mean concentration of ESAT-6 in TBM patients was significantly higher than that of the non-TBM groups. There was also a significant difference in the mean ESAT-6 expression between the confirmed TBM patients and the clinically diagnosed TBM patients (*p* < 0.01).

**Conclusions:**

Detection of ESAT-6 in the CSF of TBM patients by indirect ELISA protocol gives a reliable early diagnosis and can be used to develop an immunodiagnostic assay with increased sensitivity and specificity.

## Introduction

Tuberculosis (TB) remains a major public health problem in the twenty-first century. A third of the world’s population is infected with *Mycobacterium tuberculosis* (MTB); among them, 5–10 % of the infected population will develop the disease during their lifetime, and TBM affects about 7–12 % [1] of all tuberculosis patients. However, about 30 % of patients with TBM will die despite anti-tuberculosis chemotherapy [2]. TB is responsible for >2 million deaths per year worldwide. The situation is exacerbated by co-infection with human immunodeficiency virus (HIV).

The diagnosis of TBM relies on the isolation of MTB from CSF, but direct staining of CSF for acid-fast bacilli has very low sensitivity (10–20 %) [3]; bacilli cultures usually take 4–6 weeks and also lack sensitivity (25–70 %) [4, 5]. Yet, timely diagnosis and treatment will have a direct influence on the prognosis. So, new, affordable, sensitive, specific diagnostic assays are required.

In the present study, we used the method of indirect ELISA to detect the mycobacterial antigen ESAT-6. ESAT-6 is an early secreted and low molecular weight protein of MTB, and exists mainly in pathogenic mycobacteria but not in all *M. bovis* BCG strains or in most environmental mycobacterials examined so far [6]. Previous studies on ESAT-6 concentrated mainly on specific T cell responses and gamma interferon (IFN-γ) induced by ESAT-6. To our knowledge, there are no reports on the quantitative detection of ESAT-6 in the CSF of TBM patients.

## Methods

### Study subjects

We selected 100 patients from an inpatient department (73 males, 27 females, within the ages of 13–68 years); 10 had confirmed TBM (the direct staining of the CSF for acid-fast bacilli and/or cultures for bacilli were positive), 40 were diagnosed by clinical symptoms based on the criterion of Thwaites [5]. All cases above were diagnosed in <2 weeks after the onset of their disease, and the cases were more severe in the confirmed TBM group; four with active pulmonary tuberculous, four with tuberculoma, and two with spinal cord involvement. Non-TBM diseases (*n* = 50) were selected for the control, including multiple sclerosis (*n* = 10), acute myelitis (*n* = 10), viral meningitis (*n* = 10), purulent meningitis (*n* = 8), cryptococcal meningitis (*n* = 2), and healthy controls (*n* = 10). Samples were collected from all study groups for which patient’s consent and the ethical committee’s approval was obtained.

### Antigen and antibody

The recombinant pure antigen ESAT-6 (standard product) was bought from ProSpec (USA). The primary mAb against ESAT-6 and the secondary mAb (peroxidase conjugate goat anti-mouse IgG) were bought from Abcam (UK).

### Specimens

CSF was obtained from the 100 patients by puncture as soon as they were diagnosed and stored at −80 °C until it use.

### Establishing a standard curve

**Fig. 1:**
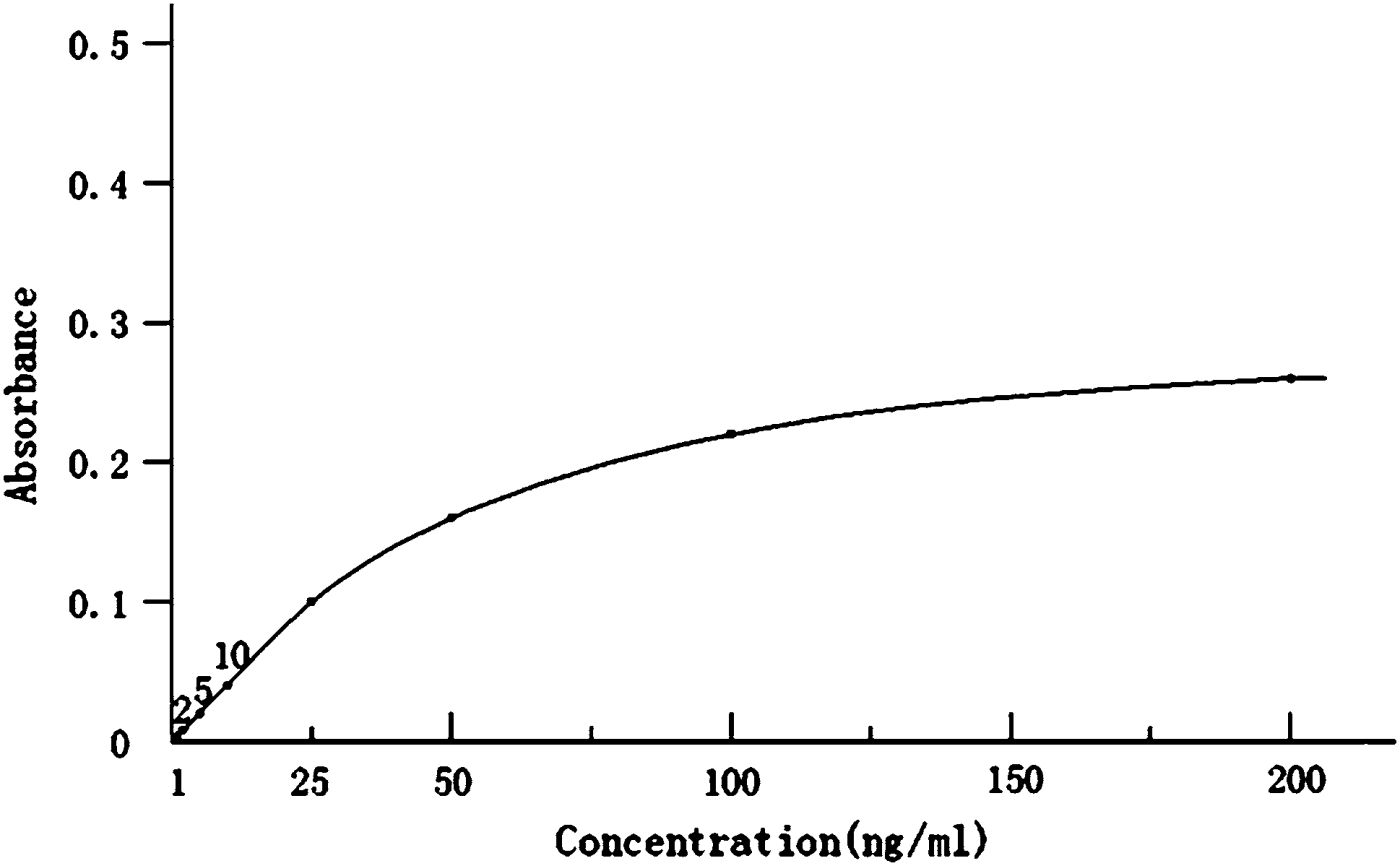
The standard curve of ESAT-6 antigen in CSF by the ELISA protocol developed in this study

Prior to patient sampling, the assay was standardized by incubating purified ESAT-6 with the mAb against ESAT-6 at different dilutions, using the indirect ELISA method (see the method in following text) to detect the absorbance of the standard product at different dilutions and to establish a standard curve, with concentration on the *X* axis vs. absorbance on the *Y* axis (Fig. 1). The different dilutions of the standard product were prepared by diluted in coating buffer (3.03 g Na_2_CO_3_, 6.0 g NaHCO_3_, 1,000 ml distilled water, pH 9.6) at 1, 2, 5, 10, 25, 50, 100, and 200 ng/ml.

### Indirect ELISA protocol

CSF samples in a volume of 50 μl (the same as the standard product) from TBM patients and control subjects were added to each well, and incubated for 2 h at room temperature. Then, the plate was washed twice by filling the wells with 20 μl of phosphate-buffered saline (PBS). The remaining protein-binding sites in the wells were blocked by adding 200 μl of 5 % non-fat dry milk per well, then incubated overnight at 4 °C and washed five times with 200 μl of PBS. The primary mAb (1:3,200, diluted in 5 % non-fat dry milk) was added (100 μl) in each well and incubated for 2 h at room temperature in the dark. The wells were then washed five times with 200 μl of PBS. The secondary antibody (1:5,000, diluted in 5 % non-fat dry milk; 100 μl) was added to each well and incubated for 2 h at room temperature in the dark. Then, the plate was washed five times with 200 μl of PBS; 100 μl the SUBS-TBM substrate was added to the wells and incubated at room temperature for 15 min, also in the dark. The reaction was then stopped with 100 μl of 0.3 M H_2_SO_4_ (stopping buffer) after sufficient color development. The absorbance of each well was read at 450 nm. Then, we calculated the concentration of each sample from the standard curve.

### Statistical analysis

Results are reported as mean ± standard deviation; differences between the TBM patient groups and controls and between the confirmed TBM group and the clinically diagnosed group were analyzed by *t* tests using SPSS software (version 13; SPSS Inc, Chicago, IL, USA). The adopted level of statistical significance was *p* < 0.05.

## Results

The minimum antigen level that could be detected was 1 ng/ml, so patients with a level of ESAT-6 in the CSF greater than 1 ng/ml were considered positive cases. The proportions of CSF cases positive for ESAT-6 in the confirmed and clinically diagnosed TBM patients were 90 % (9/10) and 87.5 % (35/40), respectively, while the positive rate for patients in the control group was 8 % (4/50). Overall, the indirect ELISA method provided 88 % sensitivity and 92 % specificity for the diagnosis of TBM using a mAb to ESAT-6. The mean concentration of ESAT-6 in the CSF of TBM patients was 85.13 ± 18.18 ng/ml, which was significantly higher than that of the non-TBM groups (2.76 ± 1.57 ng/ml, *p* < 0.001). There was also a significant difference in the mean ESAT-6 expression between the confirmed TBM patients (112.87 ± 16.67 ng/ml) and the clinically diagnosed TBM patients (78.20 ± 10.22 ng/ml, *p* < 0.001). In the TBM patient group, the level of ESAT-6 in the CSF was >70 ng/ml in 24 cases, and 14 of them had neurological sequelae, such as headache, epilepsy, or limb weakness. Patients with an ESAT-6 level below 70 ng/ml had no neurological sequelae (Table 1).

**Table 1:** The mean concentration of ESAT-6 in CSF in each group

Group	*n*	Concentration (ng/ml)
Confirmed TBM patients	10	112.87 ± 16.67
Clinically diagnosed TBM patients	40	78.20 ± 10.22
Control	50	2.76 ± 1.57

## Discussion

Early diagnosis of TBM is still difficult; besides conventional experimental methods, immunoassays have been recently adopted. Various immunoassays for detecting antigens or antibodies in the serum or CSF of TBM patients have been developed with different sensitivities and specificities. A purified protein derivative (PPD) from MTB, i.e. a complex mixture of antigens, has long been used as a skin test for TB diagnosis. The tuberculin skin test (TST) is technically simple, but it lacks diagnostic specificity in BCG-vaccinated individuals. Another widely used diagnostic test is polymerase chain reaction (PCR) using specific primers as markers for MTB, but this test is not performed correctly in all clinical laboratories and shows variable sensitivity and specificity. Many serological assays have been tried, but nearly all have failed to improve upon the time-honored sputum smear and culture approach. Currently, it is believed that antigen detection is more sensitive than antibody detection [7], especially specific MTB antigens such as ESAT-6 and the Ag85 complex. However, the early phase of MTB infection, in patients treated with corticosteroids, or in immune-deficient TBM patients, these tests may provide false negative results when antibody detection is used, and antibody detection cannot distinguish whether it is an acute infection or a latent infection. Therefore, there has been growing interest in antigen detection to diagnose TBM [8].

Since the identification of MTB ESAT-6, there has been growing interest in the use of this antigen to diagnose TB. In previous studies, it was demonstrated that most TB patients (35–92 %) can recognize ESAT-6, while healthy unrelated controls do not [8–12]. ESAT-6 is an immunodominant T cell stimulatory antigen and is recognized by specific IFN-γ-secreting T cells present in greater numbers in patients who have an active infection compared with those who are uninfected with MTB [13]. Moreover, the numbers of specific IFN-γ-secreting T cells are significantly higher in the CSF than in the peripheral blood [14, 15]. ESAT-6-induced specific IFN-γ responses have been shown to be useful in discriminating infected individuals from healthy controls [16–18]. Consequently, the possible use of ESAT-6 as a marker of *Mycobacterium tuberculosis* infection has been proposed in regions with low TB endemicity [19].

Murakami [15] reported a case that was diagnosed with TBM based on the detection of ESAT-6-specific IFN-γ production in CSF by ELISPOT (an enzyme-linked immunospot assay). Mathai [20] reported that detecting PPD antigen in CSF by Dot-Iba (a dot-immunobinding assay) provided a sensitivity of 83.3 % and specificity of 95 %. Similarly, Kashyap [21] also detected ESAT-6 by an indirect ELISA protocol; the sensitivity was 80 % and the specificity was 86 %. Overall, these findings support our results, suggesting that the detection of ESAT-6 in CSF might be developed into a diagnostic test for TBM.

In the present study, using an indirect ELISA method, we conducted a prospective study to demonstrate ESAT-6 in the CSF of TBM patients using a mAb against the antigen. The data demonstrate that positivity for ESAT-6 in cases of confirmed and clinically diagnosed TBM patients was 90 % (9/10) and 87.5 % (35/40), respectively, while the positivity for patients in the non-TBM group was 8 % (4/50), the sensitivity of detecting ESAT-6 in CSF for diagnosing TBM was 88 % and the specificity was 92 %. The minimum antigen level that could be detected was 1 ng/ml, suggesting that the indirect ELISA method is quite sensitive. However, in the non-TBM group, four cases were positive. The reason for these false-positive results is not clear, as test error cannot be ruled out, but these levels of ESAT-6 were much lower than those in TBM patients (*p* < 0.001). Another finding is that, in the confirmed TBM group, the level of ESAT-6 was higher than in the clinically diagnosed group (*p* < 0.001). This could be that confirmed, diagnosed TBM patients showed more severe clinical manifestations; so it can be speculated that the level of ESAT-6 in CSF is higher in more severe patients. In the TBM patient group, 14 cases with a level of ESAT-6 in the CSF >70 ng/ml had neurological sequelae, but patients with a level of ESAT-6 <70 ng/ml had no neurological sequelae. Accordingly, the level of ESAT-6 in CSF can differentiate between severe infection and mild infection in TMB and could help clinicians estimate the prognosis of the disease. On the other hand, all the TBM cases in the present study were diagnosed <2 weeks after the onset of disease, and the indirect ELISA test can be completed in 24 h. Therefore, the present assay is appropriate for early diagnosis of TBM with high sensitivity and specificity.

In this study, detection of ESAT-6 in TBM patients by indirect ELISA gave a reliable early diagnosis of TBM, and the test is rapid, sensitive, cost-effective, and could be performed in any standard pathology laboratory. It could be used for developing an immunodiagnostic assay with increased sensitivity and specificity.
